# Pyorrhea Alveolaris

**Published:** 1916-09

**Authors:** J. P. Buckley

**Affiliations:** Chicago, Ill.


### PYORRHEA ALVEOLARIS.

BY DR. J. P. BUCKLEY, CHICAGO, ILL.

We have been told by Dr. Kramer, of Minneapolis, who bases his assertion upon the investigations made by Dr. Ulrich, a physician of that city, that every tooth from which you have removed the pulp should be extracted, whether the canal can be filled properly or improperly, because, he says, it is impossible to remove a pulp and fill a root without lowering the resistance of the tissues in the periapical region and bacteria *will*—he does not say "may," but *it will* come along surely through the medium of the blood stream by what is known as hematolysis, and infection will always ensue.

Dr. Eisen, of Milwaukee, made the statement at Chicago, in a discussion of Dr. Callahan's paper, that every tooth which contained a gangrenous pulp, where you could not subsequently to the filling of the root perform the operation of apicoectomy, should be extracted. Now, here we have two conscientious men of large experience: one advocating the extraction of all teeth from which it is necessary to remove the pulp, and the other advocating the extraction of all teeth, under certain limitations.

In the treatment of pyorrhea it is absolutely essential, if you are going to be successful in handling the condition, that the patient know something about what is to be expected from the treatment. Sometimes it is best, when undertaking a piece of bridge work or making a plate or inserting a filling or an inlay, not to explain the details of what you expect to attain by your operation, because it may frequently be necessary or advisable to change the details of the work as it proceeds, and it may be difficult or embarrassing to be obliged to take the time to explain why we are making the change. But in the treatment of pyorrhea alveolaris, to my mind, it is essential at the beginning that the patient understand what you expect to accomplish by the treatment.

When patients are suffering from pyorrhea they are not so much interested in the fact that you can compress pus from the different pyorrheal pockets about their teeth as they are in the fact that some of their teeth are beginning to get loose and the gums are beginning to recede and expose the roots of the teeth, and the first question they ask is whether or not you can cure the condition.

I prefer to call pyorrhea alveolaris a *condition* rather that a *disease,* because I believe it is in the majority of instances a local manifestation of some constitutional disorder. That word "cure" for me has a relative meaning, and I explain at the outset what *cure* means as applied to the case presented. I tell the patient that, with the exception of such teeth as after examination I may find hopelessly diseased, a cure means that I can put the rest of the teeth in a healthy, useful and comfortable condition. If they expect me to restore all of the lost alveolar process, meaning thereby the bone surrounding the teeth holding them firmly to the jaw, or to restore gums which have receded and bring them back to the place they once were normally, or to tighten all teeth which are loosened, after my treatment, without the adjustment of some retaining appliance, then I can not cure that case, because neither I nor anyone else can do those things. I want the patient to understand clearly what I mean by a cure. I can not restore either soft or osseous tissue that had been lost.

There is no condition that you will be called upon to treat wherein it is so necessary to have the full confidence of the patients as in handling these pyorrheal cases, and I do not know of anything that will cause a patient to lose confidence more than if we undertake the treatment of a tooth so affected with pyorrhea, treat it for two or three months, perhaps a year, and at the end of that period have to acknowledge defeat and be compelled to extract. It will cause that patient to wonder whether or not it is going to be possible for us to put the other teeth in a healthy and comfortable condition, which it may be a comparatively easy thing for us to do.

We have gone quite too far in the matter of saving teeth in the last few years, that are badly abscessed, and especially where all of the alveolar process has been resorbed or badly diseased.

I can not tell always what teeth, in a given case, are hopelessly diseased. I know from my experience and observation, after having made a thorough examination of them, what teeth I believe I can put in a healthy and comfortable condition which to me means a *cure*.

If you have not been treating this condition in the past as much as you will undoubtedly be called upon to do in the future, you will be disposed to extract more teeth to begin with than you will allow yourself to do after you have become more skillful and successful with pyorrhea cases.

Dr. Rhein said that you can not treat a tooth properly, prepare the canal for the reception of the filling properly and fill the root so that the tissue in the periapical region will not become a focus of infection, unless you are reasonably paid for performing the operation. He means that you could not devitalize a pulp, remove it successfully and prepare the canal for the filling and insert that filling for the fee that we have been getting in the past. This is the usual complaint that I have heard from most dentists with whom I have discussed personally and privately this subject of the treatment of pyorrhea. They say they can not afford to give the time to the treatment of these cases for the reason the patients will not properly pay them for their services. Now I want to tell you that your patients will pay you just as much, beginning tomorrow or when you get back home next Monday, for the services you render in opening up canals of teeth and filling them, and for the proper treatment of these pyorrhea cases as they will pay you for doing any other kind of work. You should not hesitate to undertake this treatment because you hear of men in the larger cities getting larger fees in this work than you believe your patients will submit to. I do not care what fee you demand; you are entitled to as substantial a fee in the treatment of these pyorrhea cases as your patients will pay you for doing crown and bridge work.

If you can disabuse the patient's mind of the idea that you are merely cleaning teeth when you are really conscientiously curing pyorrhea, you will have advanced a long ways toward securing a greater appreciation of your services.

The treatment of pyorrhea is of three kinds. I want you to have in your mind that in the handling of a real pyorrhea case—not a mere prophylactic case, but one where you have pus pockets, where you have absorption of the alveolar process, where you have a wasting away of the gum tissue and recession of the gums, where you have a rapid or slow destruction of the pericemental membrane—you ought to consider the treatment in three different ways, and as being of three different kinds.

First and most important is the surgical; second, the medicinal, and third is the mechanical treatment.

The most important treatment of a case of pyorrhea alveolaris is to remove by surgical means and the use of surgical instruments of whatever kind, and it makes no difference to me whose or what kind of instruments you use, but by means of some kind of instrument remove all the local irritants and leave the tooth surface smooth and highly polished so that the investing tissues can rest in physiologic contact with the tooth you have just treated.

This means the removing or dressing down of all overhanging fillings of amalgam or improperly fitting inlays; it means sometimes a thing which you may hesitate to do, and that is the taking off of a crown or a bridge. You are quite ready perhaps to remove crown or bridge which another dentist has put on, but if you have placed it on the tooth you rather hesitate about taking off that local irritant, because it is something you have placed there yourself and perhaps you have caused the trouble. It does not make any difference whether that local irritant is a calcific deposit or a soft deposit, whether it is an overhanging filling impinging on the gum or a badly fitting crown or bridge, if you expect to get a good result from your surgical treatment and therapy and the following mechanical treatment it will be necessary for you first by means of your surgery to remove all those local irritants and follow that up by thoroughly polishing the tooth surface. I can not tell you how to scale a tooth. That is a thing you must work out for yourself. I can only give you the principle and you must do the work as best you know how. Dr. Rhein speaks of working hours, in one instance five hours on the crooked root of a third molar; in another instance sixteen hours to open up the canals. He finds it is hard work. I know it is hard work, but I would rather many times open down into the canals of teeth in structure which is "dead to the world," so far as the production of pain is concerned, than to scale a pyorrhea tooth, the tissues around which, unless anesthetized as I sometimes do, but seldom find it necessary to do, are sensitive. Everything that we do in dentistry is hard, if we do it conscientiously and well, and I know it is difficult in many instances to manipulate the scaler and get to the bottom of the pocket and remove all of the deposit without polishing the deposit and leaving it on, or without unnecessarily roughening the tooth surface; but it is not so much more difficult than the majority of the things you are called upon to do.

It is best, after using an instrument in a pyorrheal pocket, to have at least the point of that instrument resting in an antiseptic solution if you expect, as you will doubtless subsequently, to pick that instrument up again and use it in another pocket. There is never any excuse for using the same instrument in the same pocket a second time, because when you begin scaling in a certain pocket you should stay there until you have thoroughly scaled that tooth at that particular time with that particular instrument. But after you scale off a particular side of the tooth you want to go mesially or buccally as you go around the tooth, whichever is the best way to work, and lay it down, and you have a similar pocket in another tooth, I don't pick up that instrument and work with it down in another pocket, when that instrument has been lying on your table or on your tray for ten or fifteen minutes or even half an hour, with that pus and blood from the pocket drying upon it. The instrument being sterilized to start with, the point of it containing blood and pus should be immersed in an antiseptic solution. It makes little difference what kind of solution you employ; you can not have one strong enough to kill the germs that you have taken out of that first pyorrheal pocket and which is on the instrument during the interim I have mentioned. You would not try to have a germicide in the tray strong enough for that, but you can have one there which is of sufficient strength to hold the germ in abeyance and check its activity while the instrument is immersed in this solution, whether that solution be lysol or phenol, or any you choose to employ.

I consider the surgical procedure the most important part of your treatment for pyorrhea.

After you have thoroughly scaled the tooth by this surgical treatment you want to take sufficient time to thoroughly polish or give a thorough prophylactic treatment to the tooth you have scaled. I may scale two, three, four or six at one sitting, but whenever I have finished the surgery I am doing I always stop long enough to complete the prophylactic treatment necessary.

Two years ago, at the National meeting, Dr. Barrett, of Philadelphia, read a paper giving some details of the good work he had been doing in collaboration with Dr. Smith, and stated that they have found the amœba was present almost universally in pyorrhea pockets, as well as in certain parts of the intestinal tract, and that by the use of emetin hydrochloride they were able to report remarkable results; in fact, they felt justified in concluding from their investigation that the endamœbæ was a specific cause of pyorrhea, because this animal phagocyte, as it has been found to be, they thought caused the disease, and knowing that emetin hydrochloride was an amœbacide they announced to the world, unfortunately in the public press, though no fault of theirs, I take it, but unfortunately for us, that emetin hydrochloride was a specific for pyorrhea, with the result that we had in this country in three months more medical men treating pyorrhea than dentists. Medical men are injecting hypodermically emetin hydrochloride in the system of their patients, paying absolutely no attention to the fact that there are local irritants in the patient's mouth, asking no assistance from the dentist to remove the local irritants. They found to their sad disappointment, as well as that of the patients, that they could not cure pyorrhea in any such one-sided manner. We have not been scaling pyorrhea teeth as well as we could, we have been closing our eyes more often than we should to focal infection in the mouth, not knowing its dire results, and I will admit that we have innocently made mistakes. One of the greatest mistakes the medical profession has made is to believe that it could cure pyorrhea with emetin hydrocholoride without the all-important surgical treatment. Whether the endamœbæ happens to be in these pus pockets only incidentally, or whether the endamœbæ is a phagocyte and enjoys living upon the bacteria in the pyorrheal pockets which produces the pus, I am not here to discuss. I do say, however, regarding the medicinal treatment which may precede or follow your surgical treatment, that I believe in a certain type of these cases; in fact, I know as well as I can know anything that

I can not positively demonstrate, if you do your local surgery about the tooth thoroughly, your case will heal better under this emetin hydrochloride treatment, but it is not a specific for the disease.

We have read startling reports during the past year coming from Dr. Wright and other men who have followed up his work among the physicians of the navy where Dr. Wright, in collaboration with Dr. White, of the Navy Dental Corps, used mercuric succinimid injected hypodermically, starting with about one grain in the male, with about one-fifth grain to two-fifths grain less in the female. The results they have announced are, to say the least, startling. They do not say in the case of the eighty or ninety, or, in the recent reports, the hundred and fifty cases they treated that they had sixty-five or eighty per cent. of success, but they claim that in one hundred per cent. of cases they were curing their patients of pyorrhea by the hypodermic injection of mercuric succinimid. They also claim to have been using it long enough now so that they know that cure is permanent.

But Dr. Wright has done far better than did the medical men that injected emetin hydrochloride and let the patients go with simply that treatment. The first thing Dr. Wright did was to send his patient to the dentist in the navy, Dr. White, and after or in connection with the dentist's surgical treatment he used the medicine hypodermically. I have no right to challenge the statement of any man unless I have done the work and checked up on what he has done and honestly obtained results different from his, and I have not done that with the mercuric succinimid, and so when I make the following statement with reference to this mode of treatment, it is not because I have followed up this therapy; but I make it as based on my own experience in handling pyorrhea cases. I desire to say I can not see how the hypodermic injection of any one drug will cure all cases of pyorrhea, because I know that there are more than one constitutional disease which manifests itself in the mouth as pyorrhea alveolaris. You never saw a tubercular patient—I never did, at least, especially a pulmonary tubercular patient, one having what we used to call consumption—without pyorrhea in the mouth. If you think you did, and don't believe this statement, go to any tuberculosis sanitarium and look into the mouths of those patients and note the evidence of pyorrhea present. Dr. Rhein says that he can tell a tubercular patient by the mouth symptoms before he demonstrates the presence of the tubercular bacilli in the sputum; he also says that he can tell with absolute certainty by the mouth symptoms that a patient is suffering from Bright's disease; and I am sure that I can tell, providing the systemic disease is far enough advanced and there is sugar in the urine, by the mouth symptoms alone without urinal analysis, whether a patient has diabetes or not, because of the reddened condition of the gums, the tendency to bleed, the gums looking like a piece of fresh beef, the gums having become turgid and detached because of the presence of soft deposits and hard calculus and because of the excruciating pain associated with the condition, I am sure that I can always tell, without testing for sugar in the urine, that the patient has diabetes that is fairly well advanced. So knowing as I do that syphilis is also back of many a pyorrheal condition of the mouth, and that mercuric succinimid, properly administered, would be one of the best remedies which can be given in that systemic disorder, I can agree with the proposition that the administration of that drug might be effective in curing certain pyorrheal conditions. But knowing that there are certain other systemic diseases like those named and others involving faulty metabolism, which have their manifestations locally in the mouth, I do not see how you can expect any one remedy administered systemically or hypodermically to cure all of your pyorrhea cases. I would like to have all of you try this mercuric succinimid and note its effect, as we certainly should investigate these methods and ascertain the truth concerning them.

I will not take up your time in calling attention to this medical treatment further than to say that I believe in the use of local astringents, but I want this astringent to be used by the dentist and not by the patient. I believe that the use of an astringent mouth wash in the treatment of pyorrhea is wrong. I believe after we have scaled the pyorrhea pockets, removed the deposits and polished the teeth roots we need some astringent remedy upon that flabby and more or less congested gum tissue. The treatment given was with the idea that the gum will contract and cause a recession of that gum tissue, which I want to occur. I want the gums to be more receded and expect them to be when I get through with the treatment than before. Sometimes, if the natural healing does not cause them to recede sufficiently, or the use of the astringent remedy will not result properly, I will slit the gum, taking out a V-shaped piece.

I shall not suggest the use of any particular astringent remedy, but I want to insist that you apply it yourself and not give to the patient that remedy in solution to apply as a mouth wash.

You do not take medicine unless you are sick. If there is any tissue in the mouth in cases of pyorrhea that is sick, if you please, or diseased, it is that membrane over and around the pyorrheal pocket, and if that portion of the mucous membrane of the gum tissue is in a healthy condition it is wrong to use an astringent remedy daily as a mouth wash. It is never indicated, unless the entire mucous membrane or a large portion of it needs to be so treated.

By the mechanical treatment of pyorrhea I mean simply this: that tooth which is loose in the mouth, but you find that the tissue in the periapical region is not affected, the pocket does not extend to the end of the root—that tooth which you can scale and make healthy and comfortable for a time, you can never expect to continue to be healthy and comfortable long, because so much of the bone is absorbed that it is loose, and every time the patient closes the mouth, which is done several hundred times a day, even though the occlusion has been adjusted, which so many dentists fail to do, that tooth is prevented from tightening in the socket. That particular tooth, which you can make healthy and comfortable, but never useful, you must harness up in some way by some mechanical appliance adapted to hold it in the jaw.

In the past we have held by these mechanical appliances too many pyorrheal teeth in the mouth. You should never use a so-called retainer, or combination bridge and retainer, to supply some missing teeth and hold the loosened pyorrheal teeth in the mouth, unless you can put those teeth in such condition that the patient can keep them healthy. There are many teeth in the month which you can put in that condition and save for a number of years with your combination retainer and bridge, if you use judgment and common sense.

Pyorrhea is a condition which affects the supporting structures of teeth—the alveolar process, and in certain instances at least the pericemental membrane, and it permits an infection to take place and allows the cementum of the tooth to be saturated with pus or the end products of pathogenic germs.

We erroneously think the pericemental membrane stands only between the tooth on the one hand and the alveolar process on the other. It does more than that. It extends from the border of the alveolar process upward as far as the soft tissue is attached to the cementum of the root, and it even goes farther than that. The cementum is a connective tissue which has been calcified and arranged around the tooth in layers called lamella, and between the lamella is the lacuna, containing the cement-forming cells. The pericemental membrane is well supplied with blood vessels and nerves, extends along the border of the alveolar process. There are three divisions of that membrane: one is at the periapical region, around the apex of the tooth, the other is in the region up at the border of the bone, and the third division is where you have a fibrous connective tissue going up and holding the gum tissue in contact with the tooth structure.

The question is frequently asked, Can you regenerate bone, or can you have tissues which are once detached or separated from the tooth root reattached? You can if you put the tooth root in a healthy condition and the soft tissues remain in physiological contact with the tooth root long enough to have a new layer of cementum or lamella of the cementum deposited, into which the connective tissue fibres of the pericemental membrane are classified. It is physiologically possible, and biologically possible, to bring about this reattachment of the tooth root, but it is a very difficult thing to do in the mouth, because, while we can scale and polish and bring about a normally healthy condition of the tooth root, it is difficult to have the patient keep the tissue in physiological contact with the root long enough to have the fibres of connective tissue become classified. You never can have a reattachment in the pericemental membrane unless you have new cementum formed.

Hartzell in his discussions of this subject of pyorrhea formerly held, though I do not believe he does any more, that in this cementum we have what he called a porous substance and he told us how beautifully he scaled a tooth with what he called a "planer," and that he planed just so deep and no deeper, because if he went too far he would cut into that porous substance, and the bacteria would enter through that opening in that porous structure of the cementum and you would never be able to cure that case of pyorrhea. This caused me to wonder what Hartzell would do if called upon to scale a tooth the second time, as I sometimes have to do. If another scaling operation had to be done it would have to encroach upon that imaginary porous cementum. Noyes tells that he finds very few lacunæ in the gingival region. In the periapical region they are much more numerous. You know yourselves when you have scaled a pyorrheal tooth and have gotten the cementum of the tooth just as white and just as beautiful as was the enamel of the tooth, almost, and you did not object, neither did your patient after your scaling process, and after your prophylaxis, to the fact that the gum had receded, because the cementum of the tooth looked so nice and white.

Do not treat a pyorrheal pocket for a month or six weeks, and then wake up and find that the pulp of the tooth is dead. Ascertain whether the pulp is dead or alive, and don't treat the tooth for a long period and then discover that your pocket is extending dangerously near the end of the root, that the circulation is shut off and that the pulp of your tooth is dying. First treat that pulp before you attempt to cure the pyorrhea, if the pyorrhea is affecting that.

We sometimes have an upper molar where the disto-buccal root is practically denuded, and the lingual root you find is fairly well attached; and this is also true of the mesio-buccal. That tooth is not necessarily hopelessly diseased. It is practically solid in the jaw, the only region from which pus exudes is around the distal-root. In these cases, cut off the entire root. I want to emphasize the necessity, in many instances the necessity of slitting the gum over the pyorrheal pocket for access in scaling. When the pyorrheal pocket is not too deep, by means of astringent remedies one can assist nature in bringing about a proper recession.—*Western Dental Journal.*

